# Effect of short-term intermittent exposure to waterborne estradiol on the reproductive physiology of the round goby (*Neogobius melanostomus*)

**DOI:** 10.1007/s11356-020-09702-3

**Published:** 2020-06-22

**Authors:** Tatiana Guellard, Hanna Kalamarz-Kubiak, Bartłomiej Arciszewski

**Affiliations:** 1 Genetics and Marine Biotechnology Department, Institute of Oceanology, Polish Academy of Sciences, Sopot, Poland; 2 Prof. Krzysztof Skóra Hel Marine Station, Institute of Oceanography, Faculty of Oceanography and Geography University of Gdańsk, Hel, Poland

**Keywords:** Fish, Endocrine disruptions, 17β-estradiol, Melatonin, Thyroxine, Sex steroids, Reproductive cycle, *Neogobius melanostomus*

## Abstract

The objective of this study was to determine how the short-term exposure to a supraphysiological concentration of waterborne 17β-estradiol (E_2_) influences on melatonin (Mel) and thyroxine (T_4_) concentrations in plasma and E_2_ and 11-ketotestosterone (11-KT) concentrations in plasma and gonads in both sexes of round goby (*Neogobius melanostomus*) during the pre-spawning, spawning, late spawning and non-spawning phases. The experimental protocol was based on short-term, repeated exposures of fish to a supraphysiological dose of waterborne E_2_. Mel level was unchanged on exposure to E_2_ during the investigated phases, and its role in determining a time frame for spawning in both sexes of round goby seems to be stable in those conditions. T_4_ and sex steroids (E_2_ and 11-KT) were sensitive to the exposure of E_2_, and those changes influence gonads by accelerating oocyte development, ovulation and regression and inhibiting spermatogenesis in this species. The results demonstrate that the physiological responses of fish in all investigated phases were altered over a short window of exposure, indicating that short-term exposure to a supraphysiological dose of E_2_ may impact fish in the wild. Furthermore, round goby can be recommended as a very suitable model for studying endocrine disruptors, which is sensitive to even short exposure to E_2_.

**Electronic supplementary material:**

The online version of this article (10.1007/s11356-020-09702-3) contains supplementary material, which is available to authorized users.

## Introduction

There are several compounds entering an aquatic environment with municipal, pharmaceutical, agricultural and industrial sewage that can disturb the functioning of the endocrine system in fishes. These types of compounds belong to pollutants called endocrine disrupting compounds (EDCs). EDCs have been defined as an exogenous substance or mixture of substances that change functions of the endocrine system and provoke adverse effects in an intact organism, its offspring and also subpopulations (Vos et al. 2000). EDCs mimic endogenous hormones and this way stimulate, block or disrupt their synthesis and metabolism leading to adverse developmental, reproductive, behavioural, neurological or immune effects in organisms (reviewed in Vethaak and Legler 2012). EDCs found in the aquatic environment can be of anthropogenic as well as natural origin. EDCs naturally occurring in aquatic ecosystems include estrogens, phytoestrogens, androgens or corticosteroids (Mills and Chichester 2005; Pait and Nelson 2002). Among the group of natural estrogenic EDCs are the steroid hormones, such as 17β-estradiol (E_2_), 17α-estradiol (17α-E_2_), estrone (E_1_) and estriol (E_3_), of which E_2_ is the most potent compound (Adeel et al. 2017). Natural estrogens can act as endocrine disruptors when they are present in the environment in non-physiological concentrations. E_2_ found in aquatic environments comes mostly from domestic effluents but also from livestock waste and agriculture runoff (Desbrow et al. 1998; Ying et al. 2002; Shore and Shemesh 2003). It has been identified as one of the main components responsible for estrogenicity of sewage treatment works’ effluents (Desbrow et al. 1998). The presence of E_2_ has been reported in waste and surface waters and marine sediments in many countries, with levels ranging from a few tenths of a nanogram per litre to a few thousands of a microgram per litre (Zhang et al. 2014; Rocha and Rocha 2015; Afifi et al. 2016; Pusceddu et al. 2019). The highest and supraphysiological concentrations of E_2_ have been detected in coastal zones exposed to discharges from large urban agglomerations and runoff from animal agricultural wastes (Elnwishy et al. 2012; Zhang et al. 2014; Afifi et al. 2016).

It is known that in an organism, E_2_ is synthesized in the gonads and brain and it is the most important oestrogen crucial in neural development, growth, sexual maturation, reproduction and sexual behaviour in both female and male vertebrates, including fish (Martyniuk et al. 2006; Plant and Zeleznik 2015). Estrogens may interact with the hypothalamus–pituitary–gonad (HPG) axis by modulating steroid synthesis, transport and catabolism, and by influencing the neuroendocrine system and the involved regulatory negative and positive feedback mechanisms. Most researchers were in favour of a concept of the negative feedback of estrogens on the pituitary through direct or indirect gonadotropin-releasing hormone (GnRH) inhibition in adult fish, and a positive feedback of estrogens only in early gonadal development (reviewed in Yaron and Levavi-Sivan 2011). Nevertheless, Olivereau and Olivereau (1979) have observed positive feedback of oestrogen on the pituitary gonadotrophs in adult females of the European silver eel (*Anguilla anguilla*). In the action of estradiol, both genome (nuclear) and non-genomic (extra-nuclear) mechanisms are exploited through nuclear or membrane receptors (Falkenstein et al. 2000; Nelson and Habibi 2013). Estrogens may also exert non-genomic action through a physico-chemical interaction with the plasma membrane at only micromolar concentrations without receptor involvement (Falkenstein et al. 2000).

Effects of exogenous E_2_ have been extensively studied in fish; however, in the majority of these experiments, E_2_ has been administrated during short or long constant water exposures or via ingestion, which does not reflect the influence of environmental conditions. Doses of E_2_ used in experiments ranged from a few nanograms in water exposure to several dozen of a milligramme per kilogramme of body weight in feed (reviewed in Piferrer 2001; Mills and Chichester 2005; Dietrich and Krieger 2009). In this study, the supraphysiological dose of E_2_ was chosen on the basis of studies carried out by Hunter and Donaldson (1983), Goryczko et al. (1991), Kim et al. (1997) and Demska-Zakęś (2005) as well as based on literature concerning the occurrence of high concentrations of E_2_ in coastal zones (Elnwishy et al. 2012; Zhang et al. 2014; Afifi et al. 2016). Overall, the effects of E_2_ were noticed regardless of the length of exposure and the way the steroid was administered, and concerned, inter alia, altered plasma steroid, reduced egg or milt production, decrease in sexual behaviour and sexual characteristics in males, feminization and intersex gonads, and alterations in gonad structure (Miles-Richardson et al. 1999; Mills and Chichester 2005; Dietrich and Krieger 2009). In the natural environment, fish are exposed transiently to fluctuating levels of contaminants due to their natural diurnal and seasonal migrations linked with reproduction and also changes of feeding grounds, and due to irregular inputs of contaminants into the environment (National Research Council 1993). Only a few studies investigated intermittent waterborne E_2_ exposure (Panter et al. 2000; Martinović et al. 2008; Hyndman et al. 2010). In most studies, the applied procedures did not reflect environmental conditions. However, Panter et al. (2000) demonstrated that the response of intermittently exposed fish is more evident than that of fish exposed continuously. Therefore, this study is based on several short-term, repeated exposures of fish to a supraphysiological concentration of waterborne E_2_ using a similar protocol scheme to that used by Panter et al. (2000). The method of E_2_ administration (water baths) used in this study was selected according to Hunter and Donaldson (1983) and Goryczko et al. (1991).

The round goby (*Neogobius melanostomus*) is a batch spawning fish originating from the Ponto-Caspian basins which has successfully invaded much of the Baltic Sea and Great Lakes of North America (Kornis et al. 2012). The first occurrence of this species in the Baltic Sea was noticed in the Gulf of Gdańsk in 1990 (Skóra and Stolarski 1993). Since then, *N. melanostomus* has become one of the most abundant species in the western part of the Gulf of Gdańsk, successfully colonizing other regions of the Baltic Sea (Sapota 2012). The success of *N. melanostomus* invasion in the Baltic Sea is due to its aggressive and territorial behaviour, high adaptation to diverse environmental factors, varied diet and reproductive strategy (Kornis et al. 2012; Ojaveer et al. 2015). *N. melanostomus* was chosen as a biological model to investigate the E_2_ effects because of its sensitivity to endocrine disruption and greater predisposition to develop gonadal intersex and feminization of secondary sexual characteristics than other fish species (Bowley 2010; Guellard et al. 2015). Moreover, distribution and a large population of this invasive species in the Gulf of Gdańsk allow comprehensive monitoring without disrupting the existence of native fish populations. *N. melanostomus* is also relatively easy to catch because it is a bottom-dwelling fish occupying shallow inshore waters where it can be obtained in large numbers. To the authors’ knowledge, there is only one study investigating the influence of intraperitoneal injections of E_2_ on *N. melanostomus*, which concerned the response of vitellogenins (Vtgs) gene expression (Bowley et al. 2010). There is no complex study evaluating the effects of exogenous E_2_ on the reproductive physiology including crucial hormones such as melatonin (Mel), thyroxine (T_4_) and gonadal sex steroids in different phases of the reproductive cycle in any fish species.

The effect of Mel on estrogens was widely investigated in higher vertebrates as well as in fish because Mel synchronizes the stage of different biological rhythms, physiological processes and behavioural changes involved in reproduction (Maitra and Hasan 2016; Falcón et al. 2010). The opposite effect is not frequently studied in vertebrates except mammals. Estradiol’s effect on Mel was explored with respect to carcinogenesis and menopause, and the post-menopausal period in humans (Toffol et al. 2014; Reiter et al. 2017). T_4_ is one of the primary hormones produced by the thyroid gland and plays a major role in the regulation of metabolism, growth and development, sexual maturation and breeding cycle in all vertebrates, including fish (Leatherland 1994; Power et al. 2001). Exogenous E_2_ has been shown to alter plasma T_4_ levels in fish and in this way affecting reproductive processes (Leatherland 1985; Bandyopadhyay et al. 1991; Chakraborti and Bhattacharya 1984). However, most of the studies have used an injection of E_2_, which does not reflect environmental contaminations. E_2_ and 11-KT are the most potent steroids playing a crucial role in fish reproduction. E_2_ is a major female hormone, responsible for the development of oocytes by promoting hepatic Vtg synthesis (Lubzens et al. 2010). In males, E_2_ influences spermatogenesis, particularly spermatogonial proliferation (Schulz et al. 2010). 11-KT is crucial for stimulation of spermatogenesis and growth and development of testes (Borg 1994; Schulz et al. 2010). This male hormone is also present in low concentrations in females and it has been shown to contribute to oocyte growth (Rohr et al. 2001; Lokman et al. 2007). The effects of exogenous estrogens have been extensively studied in fish, while studies on the effect on endogenous sex steroids were less frequent. What is more, most of these studies were carried out on male or juvenile individuals (reviewed in Mills and Chichester 2005; Dietrich and Krieger 2009).

The aim of this study was to determine how the short-term, intermittent exposure to exogenous, waterborne E_2_, mimicking environmental conditions, affects Mel and T_4_ concentrations in plasma and E_2_ and 11-KT concentrations in plasma and gonads in mature females and males of *N. melanostomus* in four phases of the reproductive cycle defined as the pre-spawning, spawning, late spawning and non-spawning phases.

## Materials and methods

### Fish and experimental design

Adult females and males *N. melanostomus* (*n* = 336) were caught using fyke nets in the vicinity of Hel Harbour (54° 36′ 04.17″N, 18° 47′ 56.06″E) (Gulf of Gdańsk, southern Baltic Sea) in March, April, July and October. Captured fish were transferred to the Marine Station (Institute of Oceanography, University of Gdańsk, Poland) in Hel where they were sexed by examination of the urogenital papilla. In every studied phase, they were divided into four groups, each consisting of 21 fish of both sexes (7 females and 14 males). Each group of fish was kept at natural photoperiod and temperature (Table 1) in a 2640 L outdoor tank with a flow-through system to provide water from the Gulf of Gdańsk (~ 7 psu). In addition, hideouts made from PVC pipes were placed in each tank to mimic natural living conditions of *N. melanostomus*. Fish were fed once a day with fresh fish meat (herring), frozen mussels or shrimps. The temperature and salinity were monitored and measured using a portable meter CC-401 (ELMETRON, Zabrze, Poland) every day at noon. Before an experiment, fish were acclimatized to the tank conditions for two weeks.

**Table 1:** Average day length, water temperature and salinity (± SEM) in tanks and aquaria during investigated phases

Phase	Day length (h)	Water temperature (°C)	Salinity (psu)
Pre-spawning	13	4.8 ± 0.5	7.8 ± 0.1
Spawning	15	11.5 ± 2.5	6.7 ± 0.3
Late spawning	14	19.6 ± 1.2	6.7 ± 0.3
Non-spawning	10	11.0 ± 1.3	7.0 ± 0.1

**Fig. 1:**
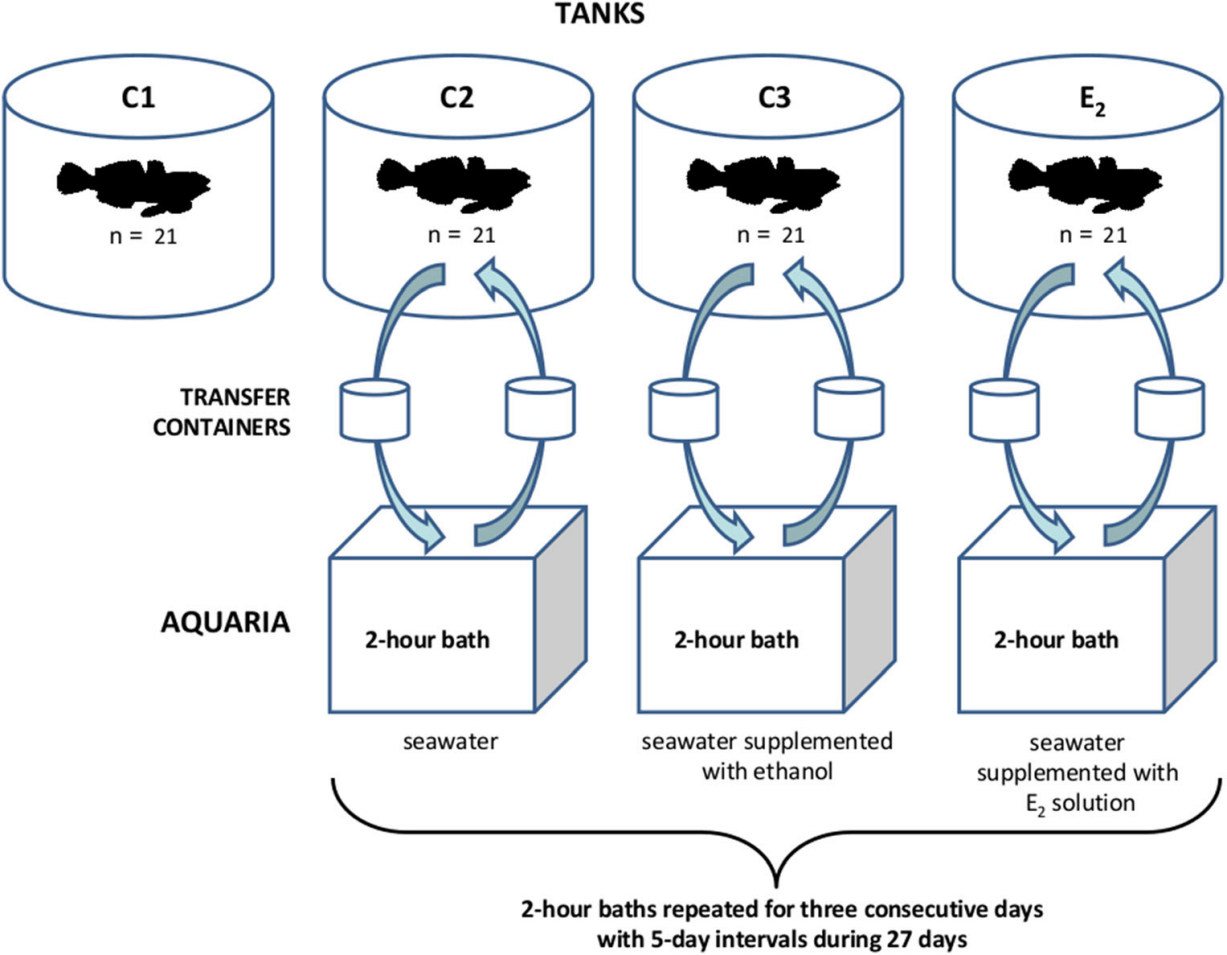
Scheme of single bath series exposures. Acclimated individuals of *N. melanostomus* were divided into the following four groups: C1—control stationary group; C2—control group transferred from its seawater tank to the seawater bath; C3—control group transferred and bathed in the seawater supplemented with ethanol; E_2_—group transferred and bathed in the seawater supplemented with E_2_ solution. Groups C2, C3 and E_2_ were subjected to four series of short-term baths with 5-day intervals for 27 days of the experiment. Each series consisted of 2-h baths repeated for three consecutive days. Each group of fish (C2, C3 and E_2_) was transferred from outdoor tanks into the appropriate indoor glass aquaria using containers with aerated water

After acclimatization, the following four experimental groups were established (Fig. 1):

C1—control stationary group kept in outdoor seawater tank that was not subjected to any kind of transfer or bath;

C2—control group transferred from its seawater tank to the seawater bath to investigate a possible influence of experimental procedure;

C3—control group transferred and bathed in the seawater supplemented with ethanol that was used as a solvent for E_2_;

E_2_—exposed group transferred and bathed in the seawater supplemented with E_2_ solution.

Groups C2, C3 and E_2_ were subjected to four series of short-term baths during the 27 days of the experiment (Fig. 1). The three-day series of baths were carried out in five-day intervals. Each bath lasted 2 h from 11 AM to 1 PM. Fish from groups C2, C3 and E_2_ were transferred in containers from outdoor tanks to indoor 500 L glass aquaria with aerated seawater. Each group was maintained in a separate aerated aquarium with the same volume of seawater, with or without supplements. The E_2_ solution was prepared before each bath by dissolving 0.05 g E_2_ (MERCK KGaA, Germany) in ethanol (J.T. Baker, Phillipsburg, USA). Nominal E_2_ and ethanol concentrations in aquaria water were 200 μg/L and 0.02%, respectively. After each two-hour bath, fish were transferred back to their outdoor tanks in containers with aerated seawater. At the beginning and at the end of each bath, water parameters (temperature, salinity, dissolved oxygen, pH, nitrate and nitrite, phosphate and ammonium/ammonia) were measured in each tank and aquarium and water samples (10 mL) from each aquarium were collected and stored at −70 °C until further E_2_ analysis. Temperature and salinity were measured using a portable meter Elmetron CC-401 (ELMETRON, Zabrze, Poland) while the other parameters (pH, dissolved oxygen, nitrate, nitrite, phosphate, ammonium and ammonia) were determined using water tests (Tetra, Spectrum Brands Company, Melle, Germany).

### Tissue sampling

At the time of sampling, fish were anaesthetized in MS-222 (tricaine methanesulfonate) water buffered solution (0.1 g/L) (Sigma-Aldrich, Saint Louis, USA). Sampling took place between 10 AM and 2 PM. Each fish was measured (to the nearest 0.1 cm) and weighed (to the nearest 0.01 g). Blood samples were collected by cardiac puncture, using a heparinized syringe. The plasma samples were centrifuged at 3000 *g* for 10 min at 4 °C and frozen at − 70 °C until analyses. After blood sampling, fish were euthanized by transection of the spinal cord. Collected gonads and liver of each fish were weighed to the nearest 0.01 g for establishing of the gonadosomatic index (GSI) and hepatosomatic index (HSI). GSI and HSI of *N. melanostomus* females and males were determined by the following equations: GSI = gonad weight / body weight × 100; HSI = liver weight / body weight × 100. Then, one gonad of each was frozen at − 70 °C and kept until steroid analysis while the second one was preserved in 4% buffered formalin (POCH, Gliwice, Poland) for histology analysis.

### Hormone measurement

#### Plasma melatonin analysis

Plasma Mel was assayed using a total melatonin radioimmunoassay (RIA) kit (RE29301, IBL International, Hamburg, Germany), with preceding extraction procedure according to the method presented by Kulczykowska et al. (2007) and modified by Guellard et al. (2019). The solid-phase extraction was carried out on Octadecyl C18 Speedisk Column, 10 μm (J.T. Baker, Phillipsburg, USA). Mel labelled with iodine-125 (^125^I) was used as a tracer for this analysis. All samples in duplicate were counted for 1 min in a Wallac Wizard 1470 gamma counter (Perkin Elmer Life Science, Waltham, USA). The detection limit was 3.0 pg/mL of plasma. The intra-assay coefficients of variation were 6.5%. The inter-assay variation was not determined because all samples were measured in the same assay.

#### Plasma thyroxine analysis

Plasma T_4_ was measured by a total thyroxine RIA kit (OCPG07-T4, Cisbio Bioassays, Codolet, France) without preceding extraction procedure according to the method presented by Kulczykowska et al. (2007). The T_4_ labelled with ^125^I was used as a tracer for RIA. All samples were assayed in duplicate and counted for 1 min in a Wallac Wizard 1470 gamma counter (Perkin Elmer Life Science, Waltham, USA). The detection limit was 1.1 ng/mL of plasma. The intra-assay coefficients of variation were 5.6%. The inter-assay variation was not determined because all samples were measured in the same assay.

#### Steroids’ analysis in plasma

Plasma E_2_ was measured using RIA kit (ESTR-CTRIA, Cisbio Bioassays, Codolet, France) without preceding extraction procedure according to the manufacturer’s protocol validated in our laboratory (Kalamarz-Kubiak et al. 2017; Guellard et al. 2019). E_2_ iodinated with ^125^I was used as a tracer. The radioactivity in each tube was measured for 1 min in a Wallac Wizard 1470 gamma counter (Perkin Elmer Life Science, Waltham, USA). The detection limit of the assay was 4.25 pg/mL. The intra-assay coefficients of variation were 2.1%. The inter-assay variation was not determined because all samples were measured in the same assay.

Plasma 11-KT was determined using a competitive enzyme immunoassay (EIA) kit (582751, Cayman Chemical, Ann Arbor, USA) with preceeding extraction procedure according to the method described by Sokołowska et al. (2013) and modified by Guellard et al. (2019). The plate was read at 412 nm using the absorbance microplate reader (Sunrise™, Tecan, Männedorf, Switzerland). All samples were assayed in duplicate. The detection limit of the assay was 0.7 pg/mL. The intra-assay coefficients of variation were 0.6%. The inter-assay variation was not determined because all samples were measured in the same assay.

#### Steroids’ analysis in gonads

Each collected ovary and testis was sonicated in 0.5 mL of phosphate buffer (0.05 M, pH 7.4) supplemented with sodium azide (0.0015 M; Sigma-Aldrich, Saint Louis, USA) using an ultrasonic homogeniser (Microson™ XL 2000, Misonix, Farmingdale, USA). Sonicated samples were centrifuged at 20,000 *g* for 20 min at 4 °C. The supernatants were stored at − 70 °C prior to the analysis of E_2_ and 11-KT levels.

The E_2_ concentrations in extracts from ovaries and testes were determined using RIA kit (ESTR-CTRIA, Cisbio Bioassays, Codolet, France) according to the method previously presented by Kulczykowska et al. (2015). Gonad supernatants (200 μL) were extracted with 1.6 mL of diethyl ether (Fisher Scientific, Loughborough, UK) according to the modified method by Mori and Kano (1984). The recovery rate of the extraction was between 86 and 109%. The detection limit of RIA was 4.25 pg/mL. The intra-assay coefficients of variation were 6.5%. The inter-assay variation was not determined because all samples were measured in the same assay.

The 11-KT concentrations in extracts from ovaries and testes were measured using EIA kit (582751, Cayman Chemical, Ann Arbor, USA) with the extraction procedure previously described by Kulczykowska et al. (2015). The recovery of extraction was between 98 and 115%. The detection limit of EIA was 1.09 pg/mL. The intra-assay coefficients of variation were 0.8%. The inter-assay variation was not determined because all samples were measured in the same assay.

#### 17β-Estradiol analysis in water

Water samples were extracted according to the methods described by Kramer et al. (1998) and Fuzzen et al. (2011) with slight modification. Samples collected from the experimental tank (5 mL) were extracted with 3 mL diethyl ether (Fisher Scientific, Loughborough, UK). Samples were vortexed for 30 s and then held at − 20 °C for 10 min to separate the layers. The diethyl ether layer was decanted into a glass tube and dried under a gentle stream of air. This extraction procedure was performed three times. Dried extracts were stored at − 20 °C until RIA analysis. The recovery rate of the extraction was between 89 and 105%. The E_2_ concentration in water samples was determined using an RIA kit (ESTR-CTRIA, Cisbio Bioassays, Codolet, France). Before measurements, extracts were reconstituted in 3 mL of phosphate buffer (0.05 M, pH 7.4) supplemented with sodium azide (0.0015 M; Sigma-Aldrich, Saint Louis, USA) and samples of 100 μL were taken for RIA analysis. A standard curve was prepared using six standard dilutions of 17, 105, 320, 880, 1910 and 4800 pg/mL. The assay was conducted in RIA tubes according to the kit manufacturer’s instructions. The tubes were vortexed for 10 s and then incubated for 2.5 h at room temperature with continuous shaking at 400 rpm. After incubation, the tubes were decanted, washed with 2 mL of distilled water and decanted again. The radioactivity of each tube was measured for 1 min using a Wallac Wizard 1470 gamma counter (Perkin Elmer Life Science, Waltham, USA). The detection limit of the assay was 17 pg/mL. The intra-assay coefficient of variation was 10.2%. The inter-assay variation was not determined because all samples were measured in the same assay.

#### Histological analysis of gonads

Gonads (ovaries and testes) preserved in 4% buffered formalin were dehydrated in ethanol and embedded in paraffin using standard histological techniques. Sections of 6 μm were cut using a semi-motorized rotary microtome (Leica RM2245, Leica Microsystems, Germany) and stained with haematoxylin and eosin. Slides from each gonad were examined with a light microscope (Leica HI1210, Leica Microsystems, Germany). The developmental stage of the gonads (ovaries and testes) was assigned according to Rocha and Rocha (2006) and Brown-Peterson et al. (2011).

### Statistical analysis

**Fig. 2:**
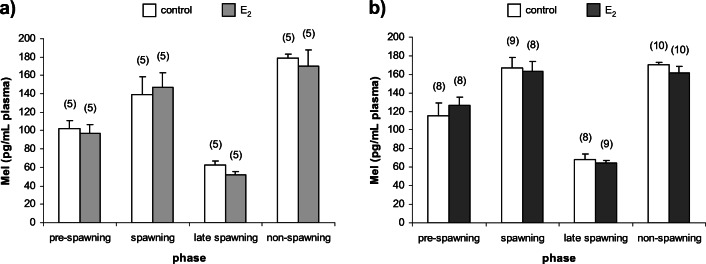
Plasma Mel concentrations in (**a**) females and (**b**) males of *N. melanostomus* after exposure to waterborne E_2_ in different phases of the reproductive cycle. Data are presented as mean ± SEM. The number of individuals is presented in brackets above each bar

**Fig. 3:**
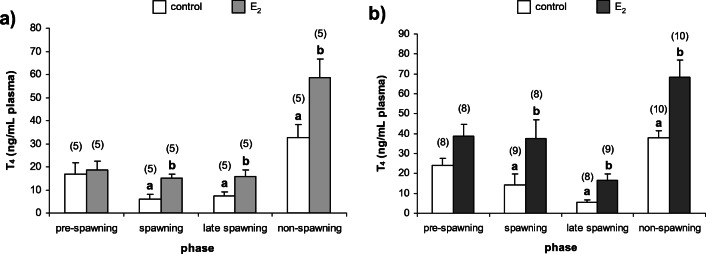
Plasma T_4_ concentrations in (**a**) females and (**b**) males of *N. melanostomus* after exposure to waterborne E_2_ in different phases of the reproductive cycle. Data are presented as mean ± SEM. The number of individuals is presented in brackets above each bar. Significant differences between the control group and the group exposed to E_2_ for each of the reproductive phases are given as letters (*p* < 0.05)

**Fig. 4:**
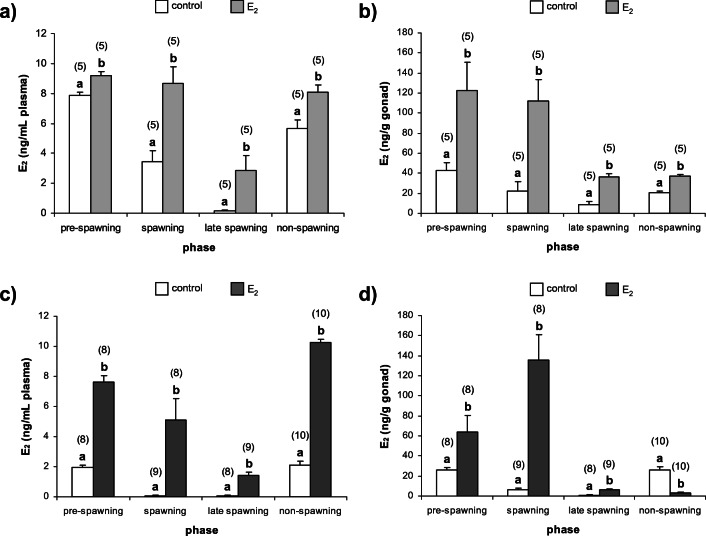
Concentrations of E_2_ in *N. melanostomus* females (**a**) plasma, (**b**) gonads and males (**c**) plasma, (**d**) gonads after exposure to waterborne E_2_ in different phases of the reproductive cycle. Data are presented as mean ± SEM. The number of individuals is presented in brackets above each bar. Significant differences between the control group and the group exposed to E_2_ for each of the reproductive phases are given as letters (*p* < 0.05)

**Fig. 5:**
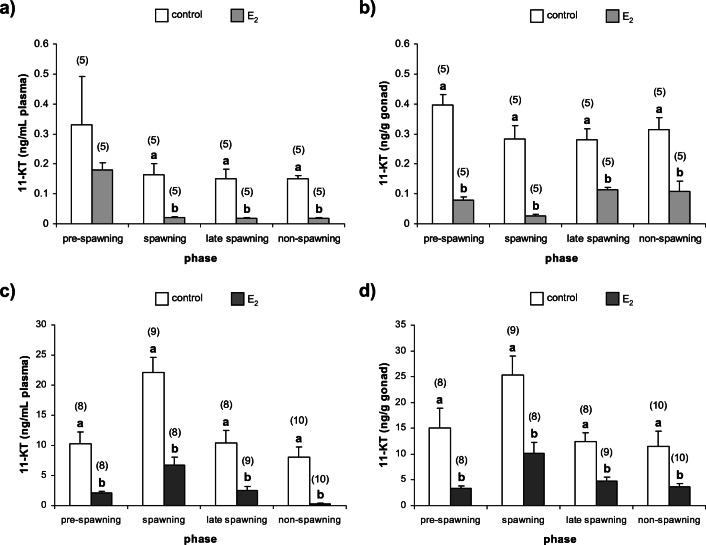
Concentrations of 11-KT in *N. melanostomus* females (**a**) plasma, (**b**) gonads and males (**c**) plasma, (**d**) gonads after exposure to waterborne E_2_ in different phases of the reproductive cycle. Data are presented as mean ± SEM. The number of individuals is presented in brackets above each bar. Significant differences between the control group and the group exposed to E_2_ for each of the reproductive phases are given as letters (*p* < 0.05)

Statistical analyses of data were carried out using STATISTICA 13, StatSoft software. The data were expressed as mean ± standard error of the mean (± SEM). Significance was accepted at *p* < 0.05. For multiple comparisons of hormone concentrations (Mel, T_4_, E_2_ and 11-KT) and GSI and HSI values among control groups during all investigated phases, one-way analysis of variance (ANOVA) was performed. There were no statistically significant differences between control groups (C1, C2 and C3) in the case of all of the investigated hormones and indexes during all investigated phases (Online Resource 1). Thus, the solvent control group (C3) was used for further analyses as a control group. For comparison of hormones’ concentrations between control and E_2_-exposed groups during all investigated phases, unpaired Student’s *t* test was performed (Figs. 2, 3, 4 and 5).

## Results

### Determination of 17β-estradiol concentrations in water

The concentrations of E_2_ in the water of control groups’ aquaria (groups C1, C2 and C3) were under the limits of detection, while the mean measured concentrations of E_2_ in aquaria of the E_2_-exposed group were 145.92 ± 0.94 μg/L at the beginning and 141.39 ± 1.43 μg/L at the end of each bath exposure in all of the investigated phases.

### Determination of environmental parameters

During the examined phases, each group of fish was kept at a natural photoperiod, temperature and salinity (Table 1). Throughout all phases, the pH of water was 7.5, dissolved oxygen concentration remained > 10 mg/L, nitrate < 12.5 mg/L, nitrite < 0.025 mg/L, phosphate < 0.02 mg/L and the concentration of ammonium and ammonia remained < 0.25 mg/L. During each short-term bath, the parameters of water were the same in the outdoor tank as well as in indoor aquaria.

### Hormone concentrations in response to 17β-estradiol exposure

#### Plasma melatonin

There were no statistically significant differences in plasma Mel concentrations between control and E_2_-exposed groups in both sexes of *N. melanostomus* in all of the phases (Fig. 2a and b).

#### Plasma thyroxine

In *N. melanostomus* females and males, there were no statistically significant differences in concentrations of plasma T_4_ in pre-spawning phase, while in other phases, plasma T_4_ concentrations were significantly increased in response to E_2_ exposure (*p* < 0.05) (Fig. 3a and b).

#### Plasma and gonadal 17β-estradiol

In *N. melanostomus* females, the plasma and gonadal E_2_ concentrations were significantly elevated (*p* < 0.05) in response to exogenous E_2_ exposure in all of the phases (Fig. 4a and b). In males, plasma and gonadal E_2_ concentrations were significantly increased (*p* < 0.05) in response to exogenous E_2_ exposure in pre-spawning, spawning and late spawning phases. In the non-spawning phase, a statistically significant increase (*p* < 0.05) in plasma E_2_ and decrease (*p* < 0.05) in gonadal E_2_ concentrations were noticed in males (Fig. 4c and d).

#### Plasma and gonadal 11-ketotestosterone

In *N. melanostomus* females, both plasma and gonadal 11-KT concentrations significantly decreased (*p* < 0.05) in response to E_2_ exposure in the spawning, late spawning and non-spawning phases (Fig. 5a and b). Similar results were obtained in plasma 11-KT concentrations in pre-spawning phase, but differences were not statistically significant. In males, plasma and gonadal 11-KT concentrations significantly decreased (*p* < 0.05) in response to exogenous E_2_ exposure in all of the investigated phases (Fig. 5c and d).

### GSI and HSI values

Statistically significant differences in GSI between control group and E_2_-exposed group (*p* < 0.05) were noticed only in the non-spawning phase for females as well as for males (Table 2). In all the examined phases, there were no statistically significant differences in HSI between the control group and E_2_-exposed group in both females and males (Table 3).

**Table 2:** Mean GSI values (± SEM) for females and males of *N. melanostomus* in control group (C3) and E_2_-exposed group in different phases; significance was accepted at *p* < 0.05

GSI				
Phase	Females		Males	
	Control group	E_2_-exposed group	Control group	E_2_-exposed group
Pre-spawning	5.21 ± 0.39	6.02 ± 0.50	1.00 ± 0.10	1.01 ± 0.20
Spawning	4.37 ± 2.27	4.74 ± 0.79	1.67 ± 0.12	1.59 ± 0.19
Late spawning	2.08 ± 0.68	2.90 ± 0.87	0.65 ± 0.17	0.95 ± 0.16
Non-spawning	2.27 ± 0.12	1.37 ± 0.14^*^	0.50 ± 0.03	0.30 ± 0.02^*^

^*^Statistically significant difference between the control group and E_2_-exposed group at *p* < 0.05

**Table 3:** Mean HSI values (± SEM) for females and males of *N. melanostomus* in control group (C3) and E_2_-exposed group in different phases; significance was accepted at *p* < 0.05

HSI				
Phase	Females		Males	
	Control group	E_2_-exposed group	Control group	E_2_-exposed group
Pre-spawning	4.60 ± 0.39	5.36 ± 0.70	4.49 ± 0.40	4.05 ± 0.38
Spawning	4.91 ± 0.47	4.89 ± 0.50	3.98 ± 0.28	4.40 ± 0.26
Late spawning	6.16 ± 0.51	6.38 ± 0.50	5.10 ± 0.52	4.23 ± 0.51
Non-spawning	7.50 ± 0.28	7.23 ± 0.45	6.62 ± 0.32	5.96 ± 0.36

### Histological analysis

Histological alterations in ovaries and testes in response to E_2_ exposure vs. control groups were distinguished in the following phases of the reproductive cycle of *N. melanostomus*: pre-spawning, spawning, late spawning and non-spawning.

#### Females

**Fig. 6:**
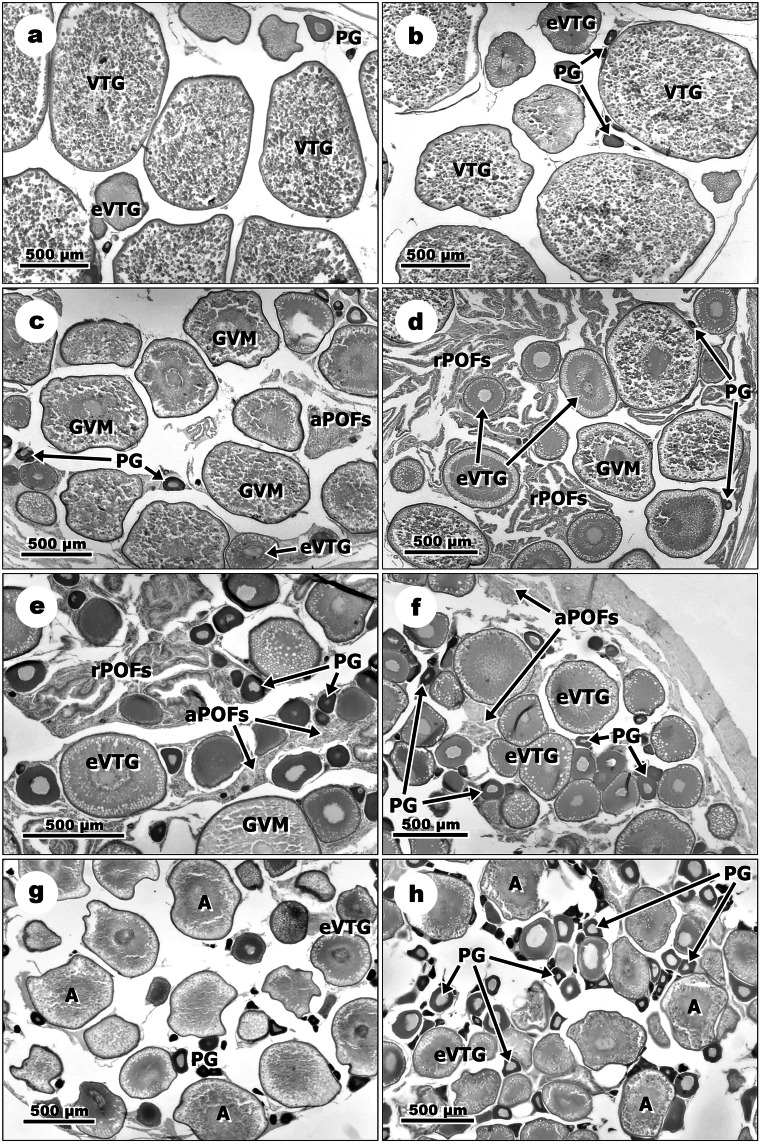
Comparison of structures of *N. melanostomus* ovaries in different phases of the reproductive cycle between control and E_2_-exposed groups. Pre-spawning phase: (**a**) Ovary of a female from the control group with the dominance of developing oocytes in the late stage of vitellogenesis (VTG) and presence of previtellogenic (PG) and early vitellogenic (eVTG) oocytes; (**b**) Ovary of a female exposed to E_2_, showing the dominance of VTG oocytes and presence of PG and eVTG oocytes. Spawning phase: (**c**) Ovary from the control group with a large number of oocytes at the early stage of germinal vesicle migration (GVM) and presence of atretic postovulatory follicles (aPOFs) and the developing batches of eVTG and PG oocytes; (**d**) Ovary of a female exposed to E_2_ with recent postovulatory follicles (rPOFs) and eVTG oocytes and sparse number of PG. Late spawning phase: (**e**) Ovary of a female from the control group with large numbers of rPOFs and aPOFs and oocytes during GVM, eVTG and PG; (**f**) Ovary of a female exposed to E_2_ showing large numbers of PG and eVTG oocytes and the presence of aPOFs. Non-spawning phase: (**g**) Regressing ovary from control group containing mostly vitellogenic oocytes in the early atresia stage (A) and eVTG and PG oocytes; (**h**) Ovary of female exposed to E_2_ showing large numbers of PG oocytes and the presence of eVTG and A oocytes. Scale bars correspond to 500 μm

In the pre-spawning phase, no histological changes were observed in females’ gonads of *N. melanostomus* exposed to E_2_ (Fig. 6a and b). Similar to the control groups, ovaries exposed to E_2_ contained mostly follicles at the late stage of vitellogenesis (VTG) and oocytes at the previtellogenic stage (PG) and early vitellogenic stage (eVTG) (Fig. 6b). In the spawning phase, the ovaries of E_2_-exposed fish contained more recent postovulatory follicles (POFs) and eVTG, and sparse number of PG oocytes in comparison to the control groups (Fig. 6c and d). The ovaries of fish from the control groups were characterized by a large number of oocytes at the early stage of germinal vesicle migration (GVM) and the presence of atretic postovulatory follicles (aPOFs) and the developing batches of eVTG and PG oocytes (Fig. 6c). In the late spawning phase, ovaries of females exposed to E_2_ in comparison to the control groups contained more PG and aPOFs but no recent postovulatory follicles (rPOFs) (Fig. 6e and f). The ovaries of *N. melanostomus* from the control groups contained large numbers of recent and aPOFs and oocytes during GVM, eVTG and PG (Fig. 6). In the non-spawning phase, exposure to E_2_ resulted in the presence of larger numbers of PG in the ovaries of round gobies than in the control groups (Fig. 6g and h). The ovaries of fish from the control groups were regressing and contained mostly vitellogenic oocytes in the early atresia stage (A) and also eVTG and PG (Fig. 6g).

#### Males

**Fig. 7:**
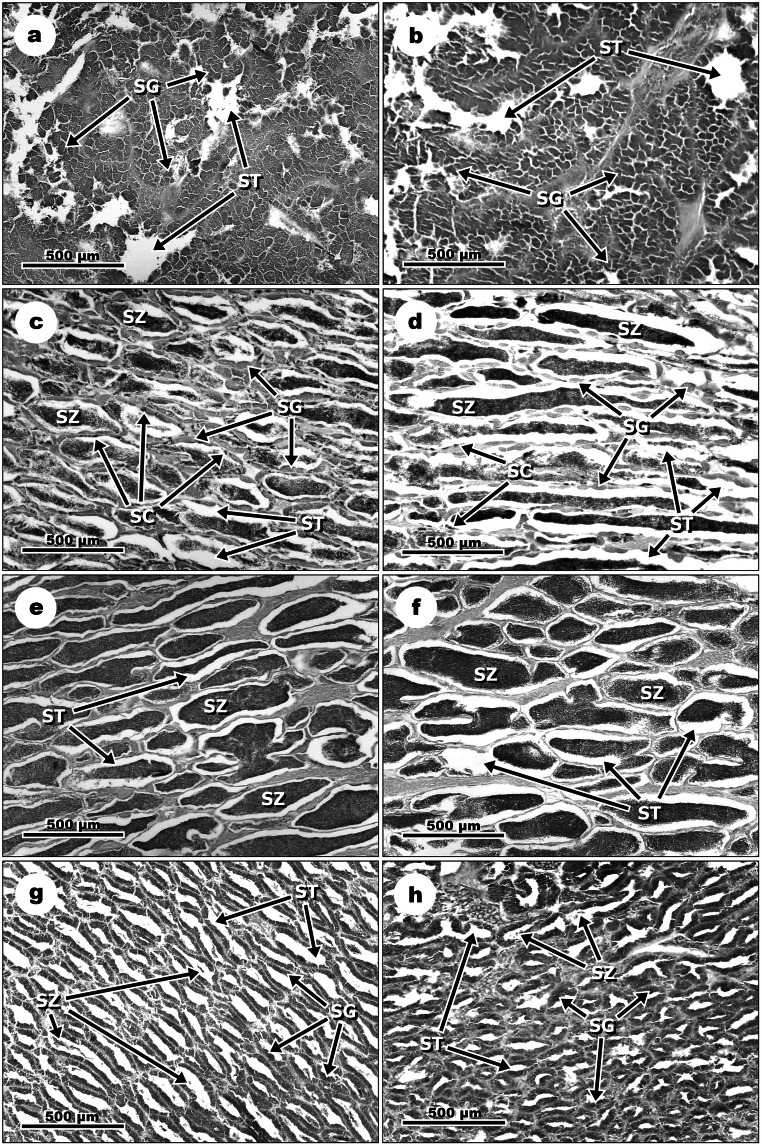
Structures of *N. melanostomus* testes in different phases of the reproductive cycle. Pre-spawning phase: (**a**) maturing testis with the dominance of spermatogonia (SG) of a male from the control group; (**b**) testis of male exposed to E_2_, with the dominance of SG. Spawning phase: (**c**) Testis of a male from the control group under active spermatogenesis with a large number of spermatozoa (SZ) and clearly visible SG and spermatocytes (SC); (**d**) Testis of male exposed to E_2_ showing low number of SG and SC in the walls of seminiferous tubules (ST) and low concentration of SZ in the lumen of ST. Late spawning phase: (**e**) fully developed testis of a male from the control group with ST filled with SZ; (**f**) testis of a male exposed to E_2_ with low concentration SZ in ST lumen. Outside spawning phase: (**g**) regressing testis with the dominance of SG and residual SZ in the lumen of ST; (**h**) testis of a male exposed to E_2_ with narrowed ST and low number of residual SZ. Scale bars correspond to 500 μm

In the pre-spawning phase, exposure to E_2_ did not cause any changes in the structure of testes. Similar to the control groups, testes of fish exposed to E_2_ were maturing and contained spermatogonia (SG)—an undifferentiated germ cell (Fig. 7a and b). In the spawning phase, exposure to E_2_ in comparison to the control groups caused a smaller number of spermatogonial cells (SG) and spermatocytes (SC) in the seminiferous tubules (ST) and less concentration of spermatozoa (SZ) in lumen of ST (Fig. 7c and d). While testes of males from the control groups were undergoing active spermatogenesis and contained a large number of SZ and clearly visible SG and SC (Fig. 7c). In the late spawning phase, exposure of *N. melanostomus* males to E_2_ resulted in less concentrated SZ in ST lumen in comparison to the control groups, where testes were fully matured and filled with SZ (Fig. 7e and f). In the non-spawning phase, testes of *N. melanostomus* exposed to E_2_, similar to fish from the control groups, were under regression with the dominance of SGs. However, the testes of E_2_-exposed fish had more narrowed ST and smaller number of residual SZ than testes of fish from the control groups (Fig. 7g and h).

## Discussion

This study determined how the short-term, intermittent exposure to waterborne E_2_ affects Mel and T_4_ concentrations in plasma and E_2_ and 11-KT concentrations in plasma and gonads in mature females and males of *N. melanostomus* during four phases of the reproductive cycle defined as the pre-spawning, spawning, late spawning and non-spawning phases. The fluctuations in Mel with greatest concentrations in the spawning and non-spawning phases are not changed in response to E_2_ exposure in both sexes of *N. melanostomus*. The functions of Mel in determining a time frame for spawning in both sexes of *N. melanostomus*, mainly depending on changes in temperature and photoperiod, seem to be stable in response to the supraphysiological dose of E_2_ and pattern of exposure to this oestrogen. The effect of E_2_ on Mel is not widely investigated in fish (Bégay et al. 1994). In rainbow trout (*Oncorhynchus mykiss*), it has been investigated that E_2_ receptors (ERs) are expressed in pineal and retina and E_2_ can modulate Mel secretion from pineal cell culture (Bégay et al. 1994). On the other hand, it is possible that applied dose of E_2_ blocks changes in Mel concentration by the dopaminergic system (Popek et al. 2010; Falcón et al. 2010; Dufour et al. 2010). It is conceivable that E_2_ may act indirectly via a noradrenergic system on Mel. However, the noradrenaline control of melatonin secretion exists in some fish species and might depend on whether the photoperiodic control of Mel production by the pineal gland relies on the pineal, or the eyes, or both (Falcón et al. 2010; Migaud et al. 2007).

It was investigated that E_2_ and T_4_ are integral in affecting the gonadal tissues in both females and males of *N. melanostomus* during the timing of initiation of spawning and also the active spawning phase (Guellard et al. 2019). The present study indicates that exposure to E_2_ enhances T_4_ plasma concentrations in both sexes of *N. melanostomus*. However, in both sexes, increase in T_4_ concentration was observed in all investigated phases except pre-spawning phase. Previously, the stimulating role of E_2_ in thyroid activity of teleosts was observed in many fish species (Singh and Raizada 1979; Chakraborti et al. 1983; Bandyopadhyay et al. 1991). Estrogens could affect the thyroid gland, directly by the stimulatory effect on thyroid peroxidase activity, thyroid epithelial height, thyroidal RNA content and T_4_ level or indirectly through stimulation of thyroid-stimulating hormone production in the pituitary (Chakraborti and Bhattacharya 1984; Chakraborti et al. 1983; Leatherland 1985; Bandyopadhyay et al. 1991). It is possible that in the present study, the acceleration of female gonadal development in response to exogenous E_2_ in the spawning, late spawning and non-spawning phases is not only due to oestrogen’s action but also modulatory effect of T_4_ on fish gonads (Leatherland 1988; Raine 2011; Habibi et al. 2012). Nevertheless, the effect of E_2_ on thyroid function in fish seems to be by no means universal because there are studies in which E_2_ has been shown to have an inhibitory or no effect on the thyroid (Leatherland 1985; Cyr et al. 1988). In females of *N. melanostomus*, there was no effect of exogenous E_2_ on plasma T_4_ concentrations as well as on ovarian histology in the pre-spawning phase. The influence of E_2_ on thyroid activity has not been widely studied in teleost males, although the stimulatory effect of E_2_ was recognized in *O. mykiss* (Leatherland 1985). The interaction of oestrogen and thyroid hormones may also be explained by cross-talk or cross-regulation between reproductive and thyroidal systems through gene expression changes in nuclear ERs and deiodinase enzymes as was documented in the male goldfish (*Carassius auratus*) (Marlatt et al. 2012; Nelson and Habibi 2016).

The results presented here demonstrate that exogenous E_2_ affects sex steroid concentrations in plasma and gonads in both females and males of *N. melanostomus* during all examined phases of the reproductive cycle. In females of *N. melanostomus*, exposure to E_2_ significantly increases plasma and ovarian E_2_ concentrations independently of the investigated phases. The stimulatory effect of exogenous E_2_ on plasma E_2_ concentration was examined inter alia in mature females of *O. mykiss*, Chinese rare minnow (*Gobiocypris rarus*), three-spined stickleback (*Gasterosteus aculeatus*) and *C. auratus* (Flett and Leatherland 1989; Miles-Richardson et al. 1999; Maunder et al. 2007; Tarkhani et al. 2012). It should be mentioned that in fish, estrogenic effects may also be mediated by elevation of endogenous free plasma oestrogen levels caused by competitive binding of estrogens to sex steroid binding globulins (Tollefsen 2002). However, estrogens may influence the HPG axis primarily by modulating steroid synthesis or by regulatory feedback mechanisms. The stimulatory effect of E_2_ on gonadal E_2_ synthesis and plasma E_2_ level in fish females is implied by the increased gonadal expression of *cyp19b* the gene encoding the enzyme responsible for the conversion (aromatization) of androgens to estrogens (Halm et al. 2002; Filby et al. 2006). Exogenous E_2_ also increased gonadal expression of genes *cyp17* and *hsd 17b* encoding enzymes which, through their role in androgen production, also affect the production of estrogens (Filby et al. 2006). The E_2_ effect on 11-KT was quite opposite, reduction of this steroid concentrations in plasma and ovaries in all examined phases of the reproductive cycle. To the authors’ knowledge, there is no available information about the effect of E_2_ on 11-KT concentration in mature fish females. Nonetheless, in mature females of fathead minnow (*Pimephales promelas*), down-regulation of gonadal expression of *hsd11b* gene encoding enzyme essential in 11-KT synthesis was observed in response to 17α-ethinylestradiol and E_2_ (Filby et al. 2006, 2007). It is possible that down-regulation of expression of *hsd11b* gene may be directly responsible for the decrease in gonadal 11-KT concentration also in *N. melanostomus* females.

In *N. melanostomus* male, exposure to exogenous E_2_ significantly increased plasma E_2_ concentration during all investigated phases. The stimulatory E_2_ effect on gonadal concentrations of E_2_ was revealed in pre-spawning, spawning and late spawning phases. In the non-spawning phase, exposure to exogenous E_2_ reduced native E_2_ concentration in testes. Furthermore, the decrease in plasma and gonadal 11-KT concentrations, in response to exogenous E_2_ was observed independently of investigated phases. The stimulatory effect of E_2_ on plasma E_2_ concentrations was well established in many fish males, e.g., *O. mykiss*, *P. promelas* and *C. auratus* (Flett and Leatherland 1989; Miles-Richardson et al. 1999; Bjerselius et al. 2001). Furthermore, the exposure to exogenous E_2_ was shown to induce plasma and gonadal E_2_ increase as well as testosterone and 11-KT decrease and testicular regression and feminization (Chang et al. 1995; Condeça and Canario 1999; Yamaguchi et al. 2006). There were two potential mechanisms involved in oestrogen’s exogenous effect in fish males, first, when E_2_ induces classic biomarkers of oestrogen responses such as hepatic ER1 expression and plasma Vtg, and second when E_2_ impacts on the HPG axis through changing steroidogenic enzyme expression profiles (Filby et al. 2006; Kim et al. 2010). Although it was presumed that liver Vtg mRNA expression or Vtg accumulation in testes may correlate with changes in gonad morphology and function, and the prevalence of intersex and feminization in teleost males, the effect of estradiol on androgen synthesis via the feminization of the expression profiles for steroidogenic enzymes appears more plausible in most fish species (Trudeau et al. 1991; Govoroun et al. 2001; Halm et al. 2002; Baron et al. 2005; Filby et al. 2006). For genes encoding key enzymes in the production of androgens in males, expression of *cyp17*, *hsd11b2*, *hsd 17b* and *star* were down-regulated, suppressing the testicular steroidogenic capacity by decreasing 11-KT release in E_2_-exposed fish males (Govoroun et al. 2001; Filby et al. 2006; de Waal et al. 2009). In contrast, expression of *cyp19b* was up-regulated in E_2_-treated males (Halm et al. 2002; Filby et al. 2006). This suppression of “male” steroidogenic enzymes by estrogens may be caused via a negative feedback of E_2_ on the follicle-stimulating hormone; however, studies on *O. mykiss*, Atlantic croaker (*Micropogonias undulatus*) and red sea bream (*Pagrus major*) suggest a direct action of E_2_ on the testes, independently of gonadotropin stimulation (Govoroun et al. 2001; Baron et al. 2005; Yamaguchi et al. 2006). It is possible that the presented mechanisms involved in oestrogen’s effects may occur in *N. melanostomus* males in all investigated phases of the reproduction cycle except non-spawning. In the non-spawning phase, exogenous E_2_ exposure increased plasma E_2_ concentration and reduced E_2_ and 11-KT concentrations in testes. It is possible that a very high plasma E_2_ concentration, as a result of exposure to E_2_, may act via the negative feedback, directly on synthesis and release of GnRH within the hypothalamus and gonadotropin from the pituitary or indirectly through up-regulation of dopamine synthesis and dopamine receptor mRNA in the brain (reviewed in Dufour et al. 2010; Yaron and Levavi-Sivan 2011). However, in male *C. auratus* in the mid-recrudescence phase it was found that E_2_ did not affect luteinizing hormone secretion but acts directly on the testes probably via a short loop feedback blocking the cytochrome P450 17A1 (17α-hydroxylase—C17, 20-lyase), enzyme responsible for the formation of androstenedione in the pathway of steroidogenesis (Trudeau et al. 1991, 1993). In conclusion, it may be assumed that E_2_ has a direct action on testes and indirect action on gonadotrophins in *N. melanostomus* males.

The short exposure to a supraphysiological dose of E_2_ causes not only changes in thyroxine and sex steroids levels but also influence gonad’s histology in both sexes of *N. melanostomus*. In the pre-spawning phase in females of *N. melanostomus*, exposure to E_2_ seems to have no effect on ovarian development. Probably, *N. melanostomus* ovaries are not sensitive to an additional dose of E_2_ when their own E_2_ concentration in plasma and gonads are very high or this exposure is too short to provoke histological changes. In the spawning phase, exogenous E_2_ has accelerated ovulation and development of eVTG oocytes. Similar results have been reported in *C. auratus* and *A. anguilla* (Olivereau and Olivereau 1979; Pankhurst and Stacey 1985). In the late spawning phase, in ovaries of *N. melanostomus*, exposure to E_2_ induced PG and eVTG oocytes growth, increasing their number and stimulated atresia of POFs. In regressing ovaries of *N. melanostomus* in the non-spawning phase, exogenous E_2_ also induced PG oocytes’ growth. Furthermore, it was observed that in *N. melanostomus* as in other fish females, high concentration of E_2_ in plasma and gonads may also be caused by persisting steroidogenesis of atretic vitellogenic oocytes (Rinchard et al. 1993; Guellard et al. 2019). It is possible that exogenous estradiol strengthens not only the steroidogenesis in atretic oocytes but also in previtellogenic oocytes, accelerating their development in *N. melanostomus*.

In the pre-spawning phase in males of *N. melanostomus*, exposure to E_2_ did not affect the structure of maturing testes. In this phase, similarly to females, exogenous E_2_ seems to have no effect on gonadal development. Testes might not be sensitive to the applied dose of E_2_ because endogenous plasma and gonadal E_2_ concentrations are already high or the exposure time is not sufficient to interfere with gonadal structure. In the spawning phase, the results indicate a significant inhibitory effect of E_2_ on *N. melanostomus* spermatogenesis. Similar observations after exposure to E_2_ have been noticed in zebrafish (*Danio rerio*) and *O. mykiss* (Lahnsteiner et al. 2006; de Waal et al. 2009). In the late spawning phase, *N. melanostomus* males’ exposure to E_2_ caused partial inhibition of spermiogenesis as was observed in *O. mykiss* (Lahnsteiner et al. 2006). Furthermore, in *N. melanostomus* males as well as in males of other fish species such as Japanese huchen (*Hucho perryi*), spotted murrel (*Channa punctatus*) or gilthead seabream (*Sparus aurata*), the high concentration of E_2_ in testes in non-spawning phase have been associated with the suppression of spermatogenesis, inhibition of spermatogonial proliferation and induction of spermatogonial atresia (Amer et al. 2001; Chaves-Pozo et al. 2007; Basak et al. 2016; Guellard et al. 2019). It is possible that exogenous E_2_ together with the high level of native E_2_ might accelerate the regression of testes in *N. melanostomus*.

Significant differences in GSI between control group and E_2_-exposed group was noticed only in the non-spawning phase for both sexes. Similar results were noticed in both sexes in many fish species during development and spawning capable phases (Medda et al. 1980; Kang et al. 2002; de Waal et al. 2009). Furthermore, it should be mentioned here that the GSI is apparently a less reliable assessment of the stage of sexual maturation in batch spawners, such as *N. melanostomus* (Zeyl et al. 2014). The decrease in GSI caused by the administration of E_2_ in the non-spawning phase can be explained by progressive ovarian regression and a changing proportion between the number of atretic and developing oocytes in females and by progressive testicular regression in males of *N. melanostomus*. Moreover, in all examined phases, there were no significant differences in HSI between control group and E_2_-exposed group in both sexes. Probably this exposure to E_2_ is too short to provoke considerable liver hypertrophy or other morphological changes. No changes in HSI after exposure to E_2_ have also been reported in both sexes of Japanese medaka (*Oryzias latipes*) by Kang et al. (2002).

## Conclusion

This is the first study to determine the influence of short-term, intermittent exposure to a supraphysiological concentration of waterborne E_2_ on Mel and T4 concentrations in plasma and E_2_ and 11-KT concentrations in plasma and gonads, supported by histological analysis of gonads, in both sexes of *N. melanostomus* during the reproductive cycle.

In the present study, Mel level is unchanged in the short time exposure to a supraphysiological dose of E_2_ during investigated phases and its role in determining a time frame for spawning in *N. melanostomus* females and males seems to be stable in those conditions. It should be noted that this stable level of Mel can protect against serious histopathological changes in gonads as a consequence of E_2_ exposure. However, T_4_ and sex steroids (E_2_ and 11-KT) were sensitive to even short exposure to E_2_. It is possible that T_4_ and sex steroids are integral in affecting the gonads of *N. melanostomus* by hastening oocyte development, ovulation and regression and inhibiting spermatogenesis in response to exogenous E_2_ during the reproductive cycle. It seems that hormonal changes due to estradiol exposure cause greater disturbances in the reproduction of males than females. In males, there is shortening spawning and fertility disturbances. In turn, in females, the acceleration in oocyte development, ovulation and regression may paradoxically enhance their reproductive potential.

In conclusion, the results presented here indicate that *N. melanostomus* representing all investigated phases are sensitive to endocrine disruption caused by E_2_ and that those physiological responses were altered over a short window of exposure, indicating the potential for a supraphysiological dose of this compound to impact teleosts in coastal zones, exposed to discharges from large urban agglomerations and runoff from animal agricultural wastes. Hence, the applied procedure using intermittent waterborne E_2_ exposure mimicking environmental conditions influences the reproductive physiology of fish. *N. melanostomus* can also be recommended as a very suitable model for studying endocrine disruptors because they are sensitive to even a short exposure to E_2_.

## Electronic supplementary material

ESM 1 (PDF 336 kb)
