# Supplementary material for: Effect of short-term intermittent exposure to waterborne estradiol on the reproductive physiology of the round goby (Neogobius melanostomus)
Source: Environ Sci Pollut Res Int. 2020 Jun 22;27(29):36799–815. doi: 10.1007/s11356-020-09702-3 (PMC7456417; doi:10.1007/s11356-020-09702-3)
Supplement: Supplementary file 1 — (PDF 336 kb) [file 11356_2020_9702_MOESM1_ESM.pdf]

Supplementary material

Environmental Science and Pollution Research

Effect of short-term intermittent exposure to waterborne estradiol on the reproductive physiology of the round goby (*Neogobius melanostomus*)

Tatiana Guellard[a,*], Hanna Kalamarz-Kubiak[a], Bartłomiej Arciszewski[b]

[a] Genetics and Marine Biotechnology Department, Institute of Oceanology, Polish Academy of Sciences, Sopot, Poland

[b] Prof. Krzysztof Skóra Hel Marine Station, Institute of Oceanography, Faculty of Oceanography and Geography University of Gdańsk, Hel, Poland

[*] Corresponding author:

Tatiana Guellard

E-mail address: tguellard@iopan.gda.pl

Table 4

Mean concentrations (± SEM) of plasma Mel and T_4 and plasma and gonadal E_2 and 11-KT in control groups of *N. melanostomus* females and males in the pre-spawning phase; significance was accepted at $p < 0.05$

Hormone	Females			Males		
	C1	C2	C3	C1	C2	C3
Mel	105.3 ± 6.08	106.5 ± 5.11	102.3 ± 8.62	107.4 ± 9.78	103.9 ± 8.47	115.0 ± 14.56
T_4	16.8 ± 2.29	16.6 ± 1.87	16.69 ± 5.11	27.7 ± 5.04	27.7 ± 4.01	24.1 ± 3.46
E_2						
plasma	7.2 ± 0.25	7.3 ± 0.24	7.9 ± 0.22	2.6 ± 0.32	2.5 ± 0.30	1.9± 0.19
gonads	48.6 ± 4.34	46.1 ± 4.60	42.6 ± 8.03	24.0 ± 2.12	25.3 ± 1.86	25.7 ± 3.08
11-KT						
plasma	0.3 ± 0.11	0.3 ± 0. 09	0.3 ± 0.16	11.7 ± 2.04	10.8 ± 1.92	10.3 ± 1.91
gonads	0.3 ± 0.04	0.3 ± 0.03	0.4 ± 0.04	16.0 ± 3.14	15.6 ± 3.60	15.1 ± 3.71

Table 5

Mean concentrations (± SEM) of plasma Mel and T_4 and plasma and gonadal E_2 and 11-KT in control groups of *N. melanostomus* females and males in the spawning phase; significance was accepted at $p < 0.05$

Hormone	Females			Males		
	C1	C2	C3	C1	C2	C3
Mel	137.7 ± 13.14	126.3 ± 7.46	147.1 ± 22.78	185.4 ± 13.68	170.6 ± 12.47	161.2 ± 11.15
T_4	6.4 ± 0.58	6.8 ± 0.62	5.5 ± 2.99	17.1 ± 4.72	14.1 ± 3.11	14.4 ± 4.89
E_2						
plasma	2.6 ± 0.71	3.5 ± 1.03	3.3 ± 0.86	0.06 ± 0.03	0.03 ± 0.01	0.06 ± 0.02
gonads	14.4 ± 3.16	21.7 ± 9.59	21.3 ± 9.43	3.3 ± 0.73	5.7 ± 1.80	6.3 ± 1.89
11-KT						
plasma	0.2 ± 0.04	0.2 ± 0.03	0.1 ± 0.04	21.9 ± 2.34	19.8 ± 1.56	22.0 ± 2.53
gonads	0.3 ± 0.04	0.4 ± 0.04	0.3 ± 0.04	27.8 ± 4.25	24.6 ± 1.94	27.5 ± 3.49

Table 6

Mean concentrations (± SEM) of plasma Mel and T_4 and plasma and gonadal E_2 and 11-KT in control groups of *N. melanostomus* females and males in the late spawning phase; significance was accepted at $p < 0.05$

Hormone	Females			Males		
	C1	C2	C3	C1	C2	C3
Mel	66.4 ± 4.08	56.9 ± 1.97	62.3 ± 4.77	65.9 ± 4.10	63.3 ± 3.84	68.5 ± 5.48
T_4	5.9 ± 2.22	8.3 ± 2.16	7.4 ± 1.63	6.2 ± 1.75	6.3 ± 2.02	5.7 ± 1.16
E_2						
plasma	0.1 ± 0.03	0.1 ± 0.04	0.1 ± 0.04	0.03 ± 0.003	0.05 ± 0.02	0.03 ± 0.01
gonads	10.1 ± 2.68	10.2 ± 2.20	9.0 ± 2.49	1.1 ± 0.80	1.8 ± 1.07	1.1 ± 0.57
11-KT						
plasma	0.2 ± 0.04	0.2 ± 0.05	0.2 ± 0.03	13.2 ± 3.39	11.4 ± 3.59	10.5 ± 2.15
gonads	0.3 ± 0.02	0.3 ± 0.05	0.3 ± 0.04	14.5 ± 3.69	13.6 ± 2.55	13.6 ± 1.52

Table 7

Mean concentrations (± SEM) of plasma Mel and T_4 and plasma and gonadal E_2 and 11-KT in control groups of *N. melanostomus* females and males in the non-spawning phase; significance was accepted at $p < 0.05$

Hormone	Females			Males		
	C1	C2	C3	C1	C2	C3
Mel	162.9 ± 9.48	187.8 ± 7.72	178.9 ± 4.56	168.4 ± 11.35	179.0 ± 4.32	169.9 ± 3.30
T_4	32.6 ± 3.89	28.9 ± 4.05	32.7 ± 5.43	38.7 ± 2.75	33.3 ± 4.07	36.4 ± 3.49
E_2						
plasma	6.1 ± 0.32	5.7 ± 0.48	5.7 ± 0.56	2.4 ± 0.46	2.1 ± 0.47	2.1 ± 0.26
gonads	17.2 ± 2.91	26.1 ± 2.00	20.2 ± 1.97	24.5 ± 2.91	24.8 ± 2.19	26.2 ± 2.99
11-KT						
plasma	0.2 ± 0.05	0.2 ± 0.03	0.1 ± 0.01	10.5 ± 1.99	7.3 ± 1.94	8.0 ± 1.79
gonads	0.4 ± 0.06	0.3 ± 0.04	0.3 ± 0.04	14.8 ± 2.55	11.4 ± 3.76	11.5 ± 2.88

Table 8

Mean GSI values (± SEM) in control groups of *N. melanostomus* females and males in different phases; significance was accepted at $p < 0.05$

Phase	Females			Males		
	C1	C2	C3	C1	C2	C3
Pre-spawning	5.29 ± 0.57	5.49 ± 0.52	5.21 ± 0.39	1.12 ± 0.05	1.34 ± 0.23	1.00 ± 0.10
Spawning	3.42 ± 0.67	3.67 ± 0.25	4.37 ± 2.27	1.54 ± 0.17	1.71 ± 0.17	1.67 ± 0.12
Late spawning	2.15 ± 0.84	3.06 ± 1.23	2.08 ± 0.68	0.64 ± 0.11	0.73 ± 0.15	0.65 ± 0.17
Non-spawning	2.12 ± 0.27	2.20 ± 0.07	2.27 ± 0.12	0.53 ± 0.02	0.52 ± 0.02	0.50 ± 0.03

Table 9

Mean HSI values (± SEM) in control groups of *N. melanostomus* females and males in different phases; significance was accepted at $p < 0.05$

Phase	Females			Males		
	C1	C2	C3	C1	C2	C3
Pre-spawning	4.69 ± 0.37	5.32 ± 0.70	4.60 ± 0.39	4.60 ± 0.36	4.65 ± 0.33	4.49 ± 0.40
Spawning	4.64 ± 0.38	5.47 ± 0.43	4.91 ± 0.47	4.29 ± 0.20	3.37 ± 0.21	3.98 ± 0.28
Late spawning	7.29 ± 0.56	6.35 ± 0.55	6.16 ± 0.51	4.26 ± 0.55	4.44 ± 0.73	5.10 ± 0.52
Non-spawning	6.72 ± 0.34	7.27 ± 0.43	7.50 ± 0.28	6.85 ± 0.24	6.56 ± 0.31	6.62 ± 0.32
